# Characterization of a *bla*_KPC-3_-carrying plasmid in a clinical isolate of *Klebsiella pneumoniae* belonging to the emerging successful clone ST147

**DOI:** 10.1128/spectrum.02338-24

**Published:** 2025-05-23

**Authors:** Esther Recacha, Mercedes Delgado-Valverde, Marina R. Pulido, Elena Pérez-Nadales, Patricia Pérez-Palacios, Inés Portillo-Calderón, Juan Manuel Sánchez-Calvo, Irene Gracia-Ahufinger, Álvaro Pascual

**Affiliations:** 1 Unidad de Gestión Clínica de Enfermedades Infecciosas y Microbiología, Hospital Universitario Virgen Macarena, Sevilla, Spain; 2 Instituto de Biomedicina de Sevilla/Hospital Virgen Macarena, Universidad de Sevilla/CSIC, Sevilla, Spain; 3 Centro de Investigación Biomédica en Red en Enfermedades Infecciosas (CIBERINFEC), Instituto de Salud Carlos III 38176, Madrid, Spain; 4 Departamento de Microbiología, Facultad de Medicina, Universidad de Sevilla, Sevilla, Spain; 5 Instituto Maimónides de Investigación Biomédica de Córdoba (IMIBIC), Hospital Universitario Reina Sofía 16501, Córdoba, Spain; 6 Departamento de Química Agrícola, Edafología y Microbiología (área Microbiología), Universidad de Córdoba, Cordoba, Spain; 7 Unit of Infectious Diseases and Clinical Microbiology, Jerez de la Frontera University Hospital 16844, Jerez de la Frontera, Spain; 8 CIBER de Enfermedades Infecciosas-CIBERINFEC (CB21/13/00049), Instituto de Salud Carlos III 38176, Madrid, Spain; University of Pretoria, Pretoria, Gauteng, South Africa

**Keywords:** *K. pneumoniae*, *bla*_KPC-3_, ST147

## Abstract

*Klebsiella pneumoniae* ST147 has emerged as a successful clone able to efficiently disseminate a number of carbapenemases, including *bla*_KPC_. This study compared a representative *bla*_KPC-3_-carrying plasmid of an ST147 clone with plasmids that usually harbor *bla*_KPC-3_ belonging to the high-risk clone ST512. Five clinical isolates of KPC-3-producing *K. pneumoniae* belonging to ST147 isolated in Southern Spain were analyzed. The first ST147 isolate detected was compared with two previous isolates of *K. pneumoniae* ST512/KPC-3 and KPC-3-encoding plasmid pKpQIL as reference plasmid. Microdilution, disk diffusion, β-CARBA test, and NG-Test CARBA 5 were used for antimicrobial susceptibility analysis and phenotypic characterization of the isolates according to EUCAST guidelines. Molecular characterization was performed using pulsed-field gel electrophoresis (PFGE)-*Xba*I, sequenced (by Illumina and Oxford Nanopore) and annotated with open-source databases (CGE tools and RAST). Plasmid incompatibility groups were analyzed using PlasmidFinder; BRIG was used to compare the *bla*_KPC-3_-harboring plasmids from three of the selected clinical isolates with each other and with pKpQIL. Clinical isolates of ST147/KPC-3 showed resistance to β-lactams, fluoroquinolones, and aminoglycosides. Clinical isolates ST147/KPC-3 and ST512/KPC-3 had identical PFGE patterns in each clone. Plasmid replicon analysis showed a plasmid with the formula IncFII (K2:A-:B-) in the ST512 isolates, identical to pKpQIL; the ST147 isolates harbored IncFII (K1:A-:B-), although the genetic environment of *bla*_KPC-3_ was the same as pKpQIL, which is Tn*4401*_IS*Kpn7* upstream of *bla*_KPC-3_ and IS*Kpn6* downstream of *bla*_KPC-3_. Plasmids harboring *bla*_KPC-3_ in ST147 and ST512 are different, although differences are subtle. The genetic environment of *bla*_KPC-3_ in the different sequence types is very conservative and identical to pKpQIL.

**IMPORTANCE:**

The successful spread of *bla*_KPC_ is primarily due to the dissemination of *K. pneumoniae* isolates belonging to high-risk clonal complex 258 (ST258, ST11, and ST512); however, new clones are emerging globally as the ST147 clone. In this study, we compare the genetic environment of a representative *bla*_KPC-3_-carrying plasmid of an ST147 clone with plasmids that usually harbor *bla*_KPC-3_ belonging to the high-risk clone ST512. Plasmids harboring *bla*_KPC-3_ detected in ST512 and ST147 were different; however, the close genetic environments of *bla*_KPC-3_ in the different plasmids (ST147 and ST512) remained conserved.

## OBSERVATION

Carbapenem resistance is spreading rapidly among Enterobacterales, mainly in *Klebsiella pneumoniae*. KPC-group carbapenemases emerged in the United States in the late 1990s and became the most commonly detected carbapenemase overall (1–4).

Isolates producing KPC-group enzymes are not only resistant to most β-lactams but are also often multidrug resistant because they encode other resistance genes, making them virtually resistant to many of the currently available first-line therapy options (5–7).

There are at least 208 variants of KPC enzymes (https://www.ncbi.nlm.nih.gov/pathogens/refgene/#KPC, accessed 27 May 2024), most of which differ from the original KPC-2 and KPC-3 enzymes by less than five amino acids (8). In particular, the KPC-3 variant differs from KPC-2 by the H274Y mutation (3).

Transmission of *bla*_KPC_ genes is mainly mediated by horizontal plasmid transfer or small mobile genetic elements (mainly *Tn*4401, a Tn3-based transposon) (9, 10). Tn*4401* is 10 kb in length, delimited by two 39-bp imperfect inverted repeat sequences, and harbors *bla*_KPC_, a Tn*3* transposase gene (*tnpA*), a Tn*3* resolvase gene (*tnpR*), and two insertion sequences, IS*Kpn6* and IS*Kpn7*. Tn*4401* is often flanked by a 5-bp target-site duplication, the result of its integration. Due to the diversity in the intervening sequence between the IS*Kpn7-ist*B and *bla*_KPC_ genes, a total of eight unique *Tn*4401 isoforms (designated a to h) have been characterized: Tn*4401*a (with a 99-bp deletion in the *bla*_KPC-3_ promoter) and Tn*4401*b (with no deletion in the *bla*_KPC-3_ promoter) are the most widespread (11).

Tn*4401* is thought to originate from the Tn*3*-based *tnpA* and *tnpR* insertion upstream of *bla*_KPC_, followed by the integration of IS*Kpn6* and IS*Kpn7* downstream and upstream of *bla*_KPC_, respectively (9).

The successful spread of *bla*_KPC_ is primarily due to the dissemination of *K. pneumoniae* isolates belonging to high-risk clonal complex 258 (CC258) (ST258, ST11, and ST512), associated with the narrow host range IncFII plasmids found in both hospital and community isolates (6–8, 12–15).

Although different incompatibility (Inc) replicon groups of *bla*_KPC_-harboring plasmids have been identified in CC258 (16), the most predominant plasmid type is IncF with FII_k_ replicons, i.e., IncFII_K1_ (FIB_pKPN like_) and IncFII_K2_ (FIB_pKPQIL like_) (14). pKpQIL was the prototype of the IncFII_K2_ group and is one of the most common *bla*_KPC_-harboring plasmids, being reported in Israel, the United States, the United Kingdom, Colombia, and Italy (16). The complete nucleotide sequence of this KPC-3-encoding plasmid pKpQIL in the epidemic *K. pneumoniae* sequence type 258 was published in 2010 (17). pKPN-3 was the prototype of IncFII_K1_ and was not initially associated with *bla*_KPC_ but was considered a virulence plasmid and coresident with pKpQIL within ST258 (14).

Recent reports have indicated that certain clones, such as *K. pneumoniae* ST307 and ST147, are emerging globally as major vehicles for the spread of antimicrobial-resistant determinants (18). Outbreaks caused by KPC-3-producing *K. pneumoniae* ST147 have previously been detected in Portugal, Italy, and Turkey (19–22), but, to our knowledge, no cases have been reported in Spain.

KPC-3-producing *K. pneumoniae* was detected in the Hospital de Jerez (Southern Spain) between March 2014 and June 2016, always associated with the ST512 clone. In July 2016, another KPC-3-producing *K. pneumoniae* isolate was detected in the same hospital. After evaluation and characterization of the isolate in the Reference Laboratory of Andalusia (PIRASOA Program; http://www.iavante.es/es/programa-formacion-pirasoa), we determined that it belonged to a new clone. We hypothesized that the new clone detected, ST147, captured the same *bla*_KPC-3_-carrying plasmid present in the previous isolates of ST512.

The aim of this study was to describe the plasmid content in *K. pneumoniae* ST147/KPC-3 isolates detected in Spain by comparing a selected *bla*_KPC-3_-harboring plasmid of emerging clone ST147 detected in Spain with plasmids found in high-risk clone ST512, which usually harbors *bla*_KPC-3_, using pKpQIL as plasmid reference.

The Reference Laboratory for Antimicrobial Resistance in Andalusia (PIRASOA program, Hospital Universitario Virgen Macarena, Seville, Spain) received five clinical isolates of *K. pneumoniae* ST147/KPC-3 between July 2016 and February 2017. The first isolate detected, 2016253-ST147/KPC-3, was the first ST147 isolate harboring *bla*_KPC-3_ in our region (Andalusia) and, to our knowledge, in Spain. This ST147 isolate was selected for comparison with other *bla*_KPC-3_-harboring clinical isolates belonging to ST512 detected in hospitals in Andalusia: CHURS-000257-ST512/KPC-3, representing the first isolation of *bla*_KPC-3_ in Andalusia and Spain (2012) (23) and 2014082-ST512/KPC-3, the first *bla*_KPC-3_ detected in the same province as ST147 (2014) (Table 1).

**TABLE 1:** Major characteristics of the clinical isolates^*a*^

Isolate number	Hospital (city)	Isolation year	Source	Clone (ST)	Contig (no.)	Genetic element	Length (bp)	Plasmid replicon group	CP-Kp plasmids	Plasmid ST	Resistome	Capsular (KL) type	O antigen (O) type
CHURS-000257	Hospital Reina Sofia (Córdoba)	2012	Clinical	ST512	Contig_0	Chromosome	5,306,646	N/A	N/A	N/A	*oqxA*, *oqxB*, *bla*_SHV-11_	KL107	O1/O2v2
					Contig_1	Chromosome	20,202	N/A	N/A	N/A	Not found	KL107	O1/O2v2
					Contig_2	Plasmid	218,006	IncF	IncFII(K), IncFIB(K)	K1:A-:B-	*aph(3')-Ia*, *aadA2*		
					Contig_3		130,907	IncF	IncFIB(pQil), IncFII(K)	K2:A-:B-	*bla*_KPC-3_, *bla*_TEM-1_, *bla*_OXA-9_		
					Contig_4		59,746	ND	IncX3	ND	*bla*_SHV-11_		
					Contig_5		39,395	ND	ColRNAI	ND	*aac(6')-Ib*		
2014082	Hospital de Jerez (Cádiz)	2014	Urine	ST512	Contig_1	Chromosome	5,376,416	N/A	N/A	N/A	*oqxA*, *oqxB*, *bla*_SHV-11_	KL107	O1/O2v2
					Contig_2	Plasmid	248,000	IncX/ IncF	IncX3, IncFII(K), IncFIB(K)	K1:A-:B-	*aadA2*, *bla*_SHV-11_		
					Contig_3		118,792	IncF	IncFIB(pQil), IncFII(K)	K2:A-:B-	*bla*_KPC-3_, *bla*_TEM-1_, *bla*_OXA-9_		
					Contig_4		13,636	ND	ColRNAI	ND	*aac(6')-Ib*		
					Contig_5	Phage phiX174	5,386	ND	Not found	N/A	Not found		
2016253	Hospital de Jerez (Cádiz)	2016	Peritoneal fluid	ST147	Contig_1	Chromosome	5,357,292	N/A	N/A	N/A	*oqxA*, *bla*_SHV-11_	KL64	O1/O2v1
					Contig_2	Plasmid	192,805	IncF	IncFIB(K)(pCAV1099-114)	Not found	Not found		
					Contig_3		115,082	IncF	IncFIB(pQil), IncFII(K)	K1:A-:B-	*bla*_KPC-3_, *bla*_TEM-1_, *bla*_OXA-9_		
					Contig_4		66,900	IncF	IncFIA(HI1)	F-:A13:B-	*aac (3)-IId*, *bla*_TEM-1_		
					Contig_5		32,178	IncR	IncR	ND	*qnrB1*		

^*a*^ ND, not determined; N/A, not applicable.

Antimicrobial susceptibility was determined using commercial microdilution MicroScan WalkAway 96 Plus NMDRM1 panels (Beckman Coulter Inc., Brea, CA, USA) and ertapenem, meropenem, and imipenem disk diffusion (OXOID) in Mueller-Hinton agar. Susceptibility results were interpreted according to EUCAST 2022 v12.0 clinical breakpoints (24). The β-CARBA test (BioRad) was used to assess imipenem hydrolysis. The study of inhibitors (phenyl boronic acid, dipicolinic acid, and cloxacillin) for phenotypic characterization of carbapenemases was performed by meropenem disk diffusion (Rosco Diagnostica). Pulsed-field gel electrophoresis (PFGE) analysis of *Xba*I-digested DNA was used to determine the initial genetic relatedness (http://www.cdc.gov/pulsenet/) of the five selected isolates. Dendrograms were created with Bionumerics v8.1 software (Applied Maths, Marcy-l’Etoile, France) using the Dice coefficient and position tolerance settings of 0.8% optimization and 1% band position tolerance.

Selected clinical isolates: 2016253 (the first of the five isolates detected that was not ST512/KPC-3 clone), CHURS-000257, and 2014082 were sequenced on an Illumina platform (MiSeq), submitted to ENA (study accession numbers PRJEB53703 and PRJEB76172), and assembled using CLC Genomics Workbench software, v9.01.1 (Qiagen). The assemblies were analyzed using the ResFinder and MLSTFinder tools of the Center for Genomic Epidemiology (https://www.genomicepidemiology.org/services/) (25). PathogenWatch (https://pathogen.watch/) was used to determine capsular (KL) and O-antigen (O) types. Assembled genomes were annotated using the Rapid Annotation using Subsystem Technology (RAST) server (http://rast.nmpdr.org/). Clinical isolates 2016253 and 2014082 were sequenced using MiniON technology (Oxford Nanopore Technologies, Inc., Oxford, UK). Library preparation was performed using the Rapid Barcoding Kit (SQK-RBK004), and the raw signals stored in fast5 file format were base-called using MinKNOW v1.4.2 ONT software (filter criteria: length 1,000 bp; quality >8). Unicycler v4.8 was used to generate a hybrid genome from the MinION long reads and the Illumina short reads (26). Clinical isolate CHURS-000257 was sequenced using PacBio technology at the hospital where it was detected. The PlasmidFinder tool was used to identify the different types of plasmids found in the whole genome of the isolates (https://cge.cbs.dtu.dk/services/PlasmidFinder/), and the pMLST tool was used to characterize the plasmids harboring *bla*_KPC-3_ (https://pubmlst.org/organisms/plasmid-mlst). The BLAST Ring Image Generator (BRIG) (27) was used to compare plasmids pCHURS-000257, p2014082, p2016253, and pKpQIL. The close genetic environments of *bla*_KPC-3_, *bla*_OXA-9_, and *bla*_TEM-1_ of our plasmids were annotated using RAST (http://rast.nmpdr.org/) and ISfinder (https://www-is.biotoul.fr), and then compared with each other and with pKpQIL, as the plasmid reference. All nucleotide-based comparisons between assemblies were performed and visualized with BLAST v2.10.1 and EasyFig v2.2.5 (28). Isoform Tn*4401* was determined by comparing the *bla*_KPC-3_ promoter of the three plasmids analyzed with the *bla*_KPC-3_ promoter present in pKpQIL, using BLAST v2.10.1.

Burrows-Wheeler Aligner software v0.7.17-r1188 (29, 30) and Samtools v1.11 (31) were used to map sequencing reads to the assembled genomes and to evaluate coverage across the assemblies and then visualized using Artemis v18.1.0 (32).

The five clinical isolates belonging to ST147/KPC-3 showed resistance to third-generation cephalosporins, aztreonam, carbapenems, fluoroquinolones, and aminoglycosides (gentamicin and tobramycin). The β-CARBA test was positive, and a positive meropenem/boronic acid synergy test was observed in all of them.

PFGE-*Xba*I showed identical patterns (Fig. S1) in all five ST147/KPC-3 *K. pneumoniae* isolates, but since they had a different PFGE profile from the ST512 isolates detected earlier, the first isolate detected was selected for sequencing.

The antimicrobial resistance genes identified were associated with resistance to β-lactams (*bla*_KPC-3_, *bla*_TEM-1_, *bla*_SHV-11_), quinolones (*oqxA, oqxB, qnrB1*), and aminoglycosides [*aac(6')-Ib*, *aadA2*, *aac(3)-IId*, *aph(3')-Ia*] (Table 1). KL64/O1/O2v1 was predicted in isolate 2016253 and KL107/O1/O2v2 in isolates CHURS-000257 and 2014082.

**Fig 1:**
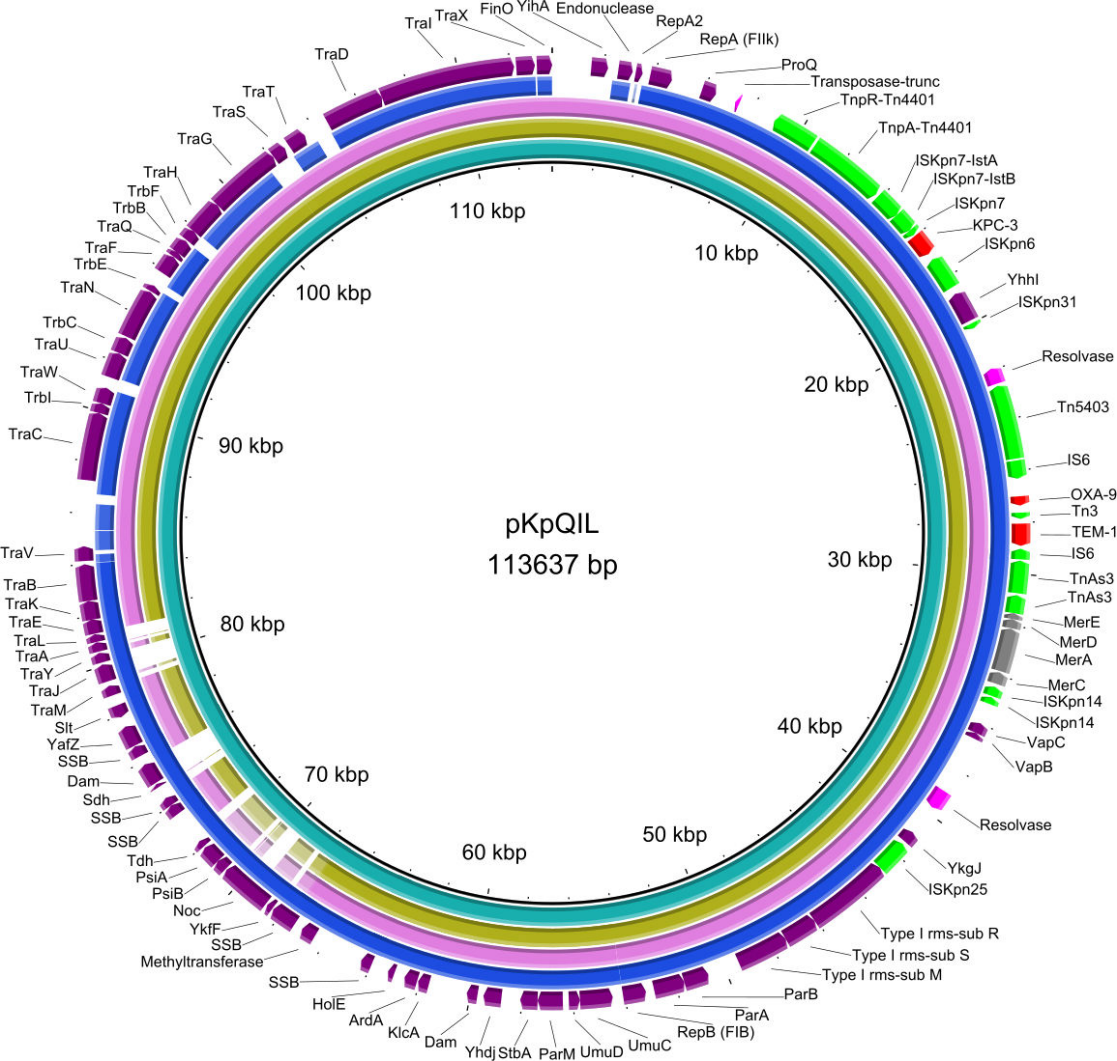
BRIG comparison of *bla*_KPC-3_-carrying plasmids pCHURS-000257 (yellow ring), p2014082 (pink ring), and p2016253 (blue ring) with the reference plasmid, pKpQIL (turquoise ring). Antibiotic and metal resistance determinants are indicated by red and gray arrows, respectively, insertion sequences by green arrows, and other plasmid components by purple arrows.

Different plasmids were found in isolates CHURS-000257 (ST512), 2014082 (ST512), and 2016253 (ST147) (Table 1). The three plasmids harboring *bla*_KPC-3_ were analyzed and then compared with pKpQIL as the reference plasmid (Fig. 1). Plasmid sizes were approximately 130.907 bp in isolate CHURS-000257, 118.792 bp in isolate 2014082, 115.082 bp in isolate 2016253, and 113.637 bp in pKpQIL. The plasmid incompatibility group found in isolates CHURS-000257 and 2014082 was IncFII (K2:A-:B-), identical to pKpQIL, whereas in isolate 2016253, the plasmid incompatibility was (K1:A-:B-). Allelic sequences 1 and 2 differed by one nucleotide at position 34 (C34G). Read coverage was around 200× in this region of the p2016253 assembly. Nucleotide identity between plasmids pCHURS-000257, p2014082, and p2016253 compared to pKpQIL using BLAST was 99%. The similarity between plasmids harboring *bla*_KPC-3_ in the two isolates (2014082 and 2016253) detected in the same province was 99%. The BRIG comparison found differences in the structure of the plasmid. Plasmid elements TraS and TrbF were present in pCHURS-000257 and p2014082 but absent in p2016253; TraA, TraJ, and TraL were present in p2016253 but absent in pCHURS-000257 and p2014082.

**Fig 2:**
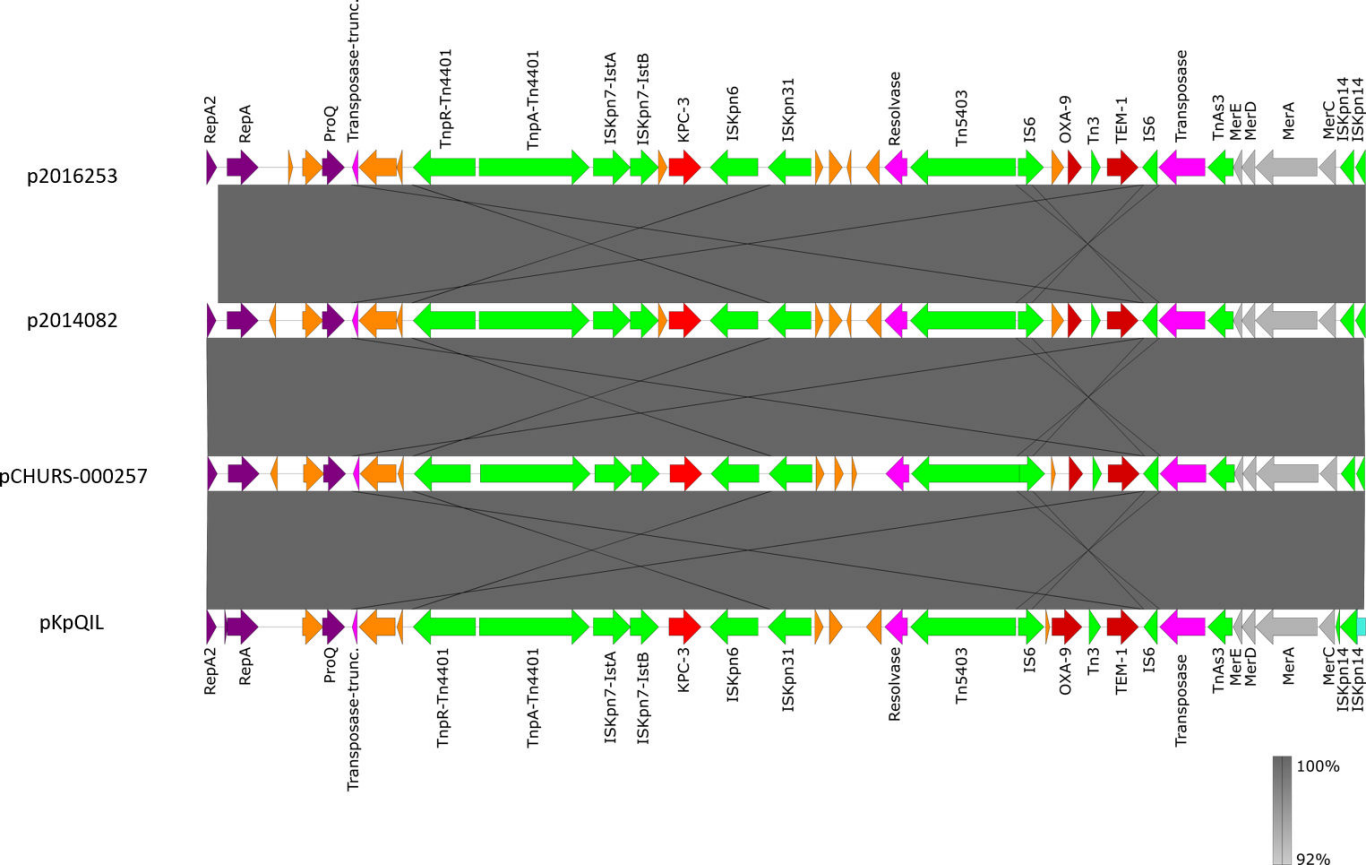
Schematic map of the genetic environment of *bla*_KPC-3_ in p2016253, pCHURS-000257, p2014082, and pKpQIL. Red arrow, β-lactamase genes; green arrow, genes related to insertion sequences; gray arrow, metal resistance determinants; purple arrow, other plasmid components; orange arrow, hypothetical proteins.

The close environment of *bla*_KPC-3_ in p2016253 (ST147) was identical to the environment of *bla*_KPC-3_ observed in pCHURS-000257 (ST512), p2014082 (ST512), and pKpQIL, described previously (9). *bla*_KPC-3_ in the analyzed plasmids was contained in Tn*4401*, with TnpR, TnpA, and IS*Kpn7* insertions upstream of *bla*_KPC-3_ and IS*Kpn6* downstream of *bla*_KPC-3_ (Fig. 2). Tn*4401* belonged to the a-isoform, with a 99-bp deletion upstream of *bla*_KPC-3_, identical to pKpQIL.

In the present study, the plasmid harboring *bla*_KPC-3_ belonging to the emerging *K. pneumoniae* clone ST147 was compared with *bla*_KPC-3_-carrying plasmids commonly detected in the high-risk CC258 found in two different hospitals in Andalusia.

The KPC-3-producing ST512 clone was identified for the first time in Spain in an Andalusian hospital in 2012 (23); since then it has been detected in other hospitals, mainly associated with high-risk clone ST258/512. In July 2016, a clinical isolate of KPC-3-producing *K. pneumoniae* ST147, a different clone type from the one harboring *bla*_KPC-3_ in Spain until then, was detected in another hospital in Andalusia. As a KPC-3/ST512 isolate had been found a month previously in the same hospital, it was thought to be associated with the previous isolate. However, genetic characterization in the Andalusian Reference Laboratory revealed a new clone, ST147, harboring *bla*_KPC-3_. In total, five clinical isolates of KPC-3-producing *K. pneumoniae* ST147, all with the same PFGE pattern and identical to the first, were detected between 2016 and 2017.

*bla*_KPC-3_ in *K. pneumoniae* is generally located on a plasmid platform in a Tn*3*-based transposon, Tn*4401*. In all isolates, the *bla*_KPC−3_ was inserted between IS*Kpn7* (upstream) and IS*Kpn6* (downstream) in a structure previously described as Tn*4401* (9). *bla*_KPC-3_ is usually carried by IncF plasmids, but allelic differences in the FIIk replicons were observed between the plasmids harboring *bla*_KPC-3_ belonging to clone ST147 (IncFII_K1_) and clone ST512 (IncFII_K2_). Plasmids harboring *bla*_KPC-3_ had 99% nucleotide similarity to pKpQIL, the reference plasmid. Comparison of the plasmids using BRIG showed structural differences. The absence of TraS in p2016253 as compared to pCHURS-000257, p2014082, and pKpQIL may alter conjugation since TraS functions to prevent redundant donor-donor conjugation (donors with identical plasmids) (33). In addition, TraA, TraJ, and TraL involved in pilin synthesis, regulation, and pilus assembly, respectively (34), were identified in p2016253 and pKpQIL but not in pCHURS-000257 and p2014082. It is not clear to what extent these modifications might affect the conjugation process.

The ST147 clone emerged in the early 1990s, and its global spread occurred during the late 1990s and early 2000s, was driven primarily by quinolone resistance-determining region mutations and the acquisition of *bla*_CTX-M-15_ located on IncF or IncR plasmids (35). Further global spread of ST147 occurred in the mid- to late 2000s, associated with the acquisition of a number of carbapenemase genes *bla*_VIM_, *bla*_KPC_, *bla*_OXA-48_, and *bla*_NDM_ in different geographic locations (18). One year before the KPC-3-producing *K. pneumoniae* ST147 clone was detected in Andalusia, a large outbreak caused by the ST147/KPC-3/K-64 lineage originated in the north of Portugal. In Portugal, ST147-K64 *K. pneumoniae*, the initial outbreak clone, remains predominant, although other high-risk clones have since emerged (19). In Spain, however, the most prevalent carbapenemase genes carried by the ST147 clone in recent years have been *bla*_OXA-48_ and *bla*_NDM_ (http://gesdoc.isciii.es/gesdoccontroller?action=download&id=08/03/20235129d36c2c; RedlabRA); *bla*_KPC-3_ is a carbapenemase carried mainly by the ST512/258 clone. The disappearance of ST147/KPC-3/K-64 contrasts with the persistence of this lineage in other regions such as Portugal.

While Tn*4401* was reported as the genetic structure mediating original *bla*_KPC_ acquisition, with the gene order of Tn*4401-tnpR*, Tn*4401-tnpA*, IS*Kpn7*, *bla*_KPC_, and IS*Kpn6*, described above, some *bla*_KPC_ variants have shown variations in Tn*4401* with IS*Kpn6* and IS*Kpn28* or IS*Kpn27* and IS*Kpn6* surrounding the carbapenemase (36). Tn*4401* isoforms with variable deletions between IS*Kpn7* and *bla*_KPC_ have also been described, conferring different promoter regions to the gene and consequently different expression levels of the *bla*_KPC_ gene, suggesting that the region surrounding *bla*_KPC_ is undergoing recombination and that Tn*4401* itself is heterogeneous and highly plastic (11, 37). Plasmids with Tn*4401*a and Tn*4401*h were more resistant to meropenem (≥16 and ≥16, respectively), ertapenem (≥8 and 4, respectively), and cefepime (≥64 and 4, respectively) than strains with Tn*4401*b (no deletion) (11). In this study, the analyzed plasmids containing *bla*_KPC-3_ were located within a mobile transposon, Tn*4401*a.

In addition to Tn*4401*, *bla*_KPC_ could be mobilized in other transposable elements, such as Tn*1721* variants and IS*26,* which play an important role in the spread of *bla*_KPC_ in Asia (36).

Clones other than CC258 have been identified harboring *bla*_KPC-3_ in different Tn*4401* isoforms, such as ST384 and ST388 in Spain (38) and ST13, ST17, ST231, and ST4446, recently described in Portugal (39).

In conclusion, our work highlights some interesting findings: the plasmids harboring *bla*_KPC-3_ detected in ST512 and ST147 were different between them and different from the archetypal pKpQIL used as a reference, although differences found were subtle. This fact refutes our initial hypothesis of uptake by the emerging clone ST147 of the same plasmid found in ST512 (the clone detected a month earlier in the same hospital). Despite the plasmid differences, the close genetic environments of *bla*_KPC-3_ in the different plasmids remained conserved, as described above (40, 41).

## SUPPLEMENTAL MATERIAL

Fig. S1 spectrum.02338-24-s0001.docx Dendrogram of pulsed-field gel electrophoresis analysis.

ASM does not own the copyrights to Supplemental Material that may be linked to, or accessed through, an article. The authors have granted ASM a non-exclusive, world-wide license to publish the Supplemental Material files. Please contact the corresponding author directly for reuse.

## References

[1] Yigit H, Queenan AM, Anderson GJ, Domenech-Sanchez A, Biddle JW, Steward CD, Alberti S, Bush K, Tenover FC. 2001. Novel carbapenem-hydrolyzing β-lactamase, KPC-1, from a carbapenem-resistant strain of Klebsiella pneumoniae. Antimicrob Agents Chemother 45:1151–1161. doi:10.1128/AAC.45.4.1151-1161.2001 11257029 PMC90438

[2] Yigit H, Queenan AM, Rasheed JK, Biddle JW, Domenech-Sanchez A, Alberti S, Bush K, Tenover FC. 2003. Carbapenem-resistant strain of Klebsiella oxytoca harboring carbapenem-hydrolyzing beta-lactamase KPC-2. Antimicrob Agents Chemother 47:3881–3889. doi:10.1128/AAC.47.12.3881-3889.2003 14638498 PMC296202

[3] Woodford N, Tierno PM Jr, Young K, Tysall L, Palepou M-FI, Ward E, Painter RE, Suber DF, Shungu D, Silver LL, Inglima K, Kornblum J, Livermore DM. 2004. Outbreak of Klebsiella pneumoniae producing a new carbapenem-hydrolyzing class A beta-lactamase, KPC-3, in a New York Medical Center. Antimicrob Agents Chemother 48:4793–4799. doi:10.1128/AAC.48.12.4793-4799.2004 15561858 PMC529220

[4] Oteo J, Pérez-Vázquez M, Bautista V, Ortega A, Zamarrón P, Saez D, Fernández-Romero S, Lara N, Ramiro R, Aracil B, Campos J, Spanish Antibiotic Resistance Surveillance Program Collaborating Group. 2016. The spread of KPC-producing Enterobacteriaceae in Spain: WGS analysis of the emerging high-risk clones of Klebsiella pneumoniae ST11/KPC-2, ST101/KPC-2 and ST512/KPC-3. J Antimicrob Chemother 71:3392–3399. doi:10.1093/jac/dkw321 27530752 PMC5890657

[5] Walther-Rasmussen J, Høiby N. 2007. Class A carbapenemases. J Antimicrob Chemother 60:470–482. doi:10.1093/jac/dkm226 17595289

[6] Munoz-Price LS, Poirel L, Bonomo RA, Schwaber MJ, Daikos GL, Cormican M, Cornaglia G, Garau J, Gniadkowski M, Hayden MK, Kumarasamy K, Livermore DM, Maya JJ, Nordmann P, Patel JB, Paterson DL, Pitout J, Villegas MV, Wang H, Woodford N, Quinn JP. 2013. Clinical epidemiology of the global expansion of Klebsiella pneumoniae carbapenemases. Lancet Infect Dis 13:785–796. doi:10.1016/S1473-3099(13)70190-7 23969216 PMC4673667

[7] Pitout JDD, Nordmann P, Poirel L. 2015. Carbapenemase-producing Klebsiella pneumoniae, a key pathogen set for global nosocomial dominance. Antimicrob Agents Chemother 59:5873–5884. doi:10.1128/AAC.01019-15 26169401 PMC4576115

[8] Bush K, Bradford PA. 2020. Epidemiology of β-lactamase-producing pathogens. Clin Microbiol Rev 33:e00047-19. doi:10.1128/CMR.00047-19 32102899 PMC7048014

[9] Naas T, Cuzon G, Villegas M-V, Lartigue M-F, Quinn JP, Nordmann P. 2008. Genetic structures at the origin of acquisition of the beta-lactamase bla KPC gene. Antimicrob Agents Chemother 52:1257–1263. doi:10.1128/AAC.01451-07 18227185 PMC2292522

[10] Shen P, Wei Z, Jiang Y, Du X, Ji S, Yu Y, Li L. 2009. Novel genetic environment of the carbapenem-hydrolyzing β-lactamase KPC-2 among Enterobacteriaceae in China. Antimicrob Agents Chemother 53:4333–4338. doi:10.1128/AAC.00260-09 19620332 PMC2764158

[11] Cheruvanky A, Stoesser N, Sheppard AE, Crook DW, Hoffman PS, Weddle E, Carroll J, Sifri CD, Chai W, Barry K, Ramakrishnan G, Mathers AJ. 2017. Enhanced Klebsiella pneumoniae carbapenemase expression from a novel Tn4401 deletion. Antimicrob Agents Chemother 61:e00025-17. doi:10.1128/AAC.00025-17 28373185 PMC5444142

[12] Mathers AJ, Peirano G, Pitout JDD. 2015. The role of epidemic resistance plasmids and international high-risk clones in the spread of multidrug-resistant Enterobacteriaceae. Clin Microbiol Rev 28:565–591. doi:10.1128/CMR.00116-14 25926236 PMC4405625

[13] Satlin MJ, Chen L, Patel G, Gomez-Simmonds A, Weston G, Kim AC, Seo SK, Rosenthal ME, Sperber SJ, Jenkins SG, Hamula CL, Uhlemann A-C, Levi MH, Fries BC, Tang Y-W, Juretschko S, Rojtman AD, Hong T, Mathema B, Jacobs MR, Walsh TJ, Bonomo RA, Kreiswirth BN. 2017. Multicenter clinical and molecular epidemiological analysis of bacteremia due to carbapenem-resistant Enterobacteriaceae (CRE) in the CRE epicenter of the United States. Antimicrob Agents Chemother 61:e02349-16. doi:10.1128/AAC.02349-16 28167547 PMC5365653

[14] Peirano G, Bradford PA, Kazmierczak KM, Chen L, Kreiswirth BN, Pitout JDD. 2017. Importance of clonal complex 258 and IncFK2-like plasmids among a global collection of Klebsiella pneumoniae with bla_KPC_. Antimicrob Agents Chemother 61. doi:10.1128/AAC.02610-16 PMC5365689 28167556

[15] Meletis G, Chatzopoulou F, Fragkouli A, Alexandridou M, Mavrovouniotis I, Chatzinikolaou A, Chatzidimitriou D. 2020. Whole-genome sequencing study of KPC-encoding Klebsiella pneumoniae isolated in Greek private laboratories from non-hospitalised patients. J Glob Antimicrob Resist 20:78–81. doi:10.1016/j.jgar.2019.07.027 31390536

[16] Chen L, Mathema B, Chavda KD, DeLeo FR, Bonomo RA, Kreiswirth BN. 2014. Carbapenemase-producing Klebsiella pneumoniae: molecular and genetic decoding. Trends Microbiol 22:686–696. doi:10.1016/j.tim.2014.09.003 25304194 PMC4365952

[17] Leavitt A, Chmelnitsky I, Carmeli Y, Navon-Venezia S. 2010. Complete nucleotide sequence of KPC-3-encoding plasmid pKpQIL in the epidemic Klebsiella pneumoniae sequence type 258. Antimicrob Agents Chemother 54:4493–4496. doi:10.1128/AAC.00175-10 20696875 PMC2944570

[18] Peirano G, Chen L, Kreiswirth BN, Pitout JDD. 2020. Emerging antimicrobial-resistant high-risk Klebsiella pneumoniae clones ST307 and ST147. Antimicrob Agents Chemother 64:e01148-20. doi:10.1128/AAC.01148-20 32747358 PMC7508593

[19] Guerra AM, Lira A, Lameirão A, Selaru A, Abreu G, Lopes P, Mota M, Novais Â, Peixe L. 2020. Multiplicity of carbapenemase-producers three years after a KPC-3-producing K. pneumoniae ST147-K64 hospital outbreak. Antibiotics (Basel) 9:806. doi:10.3390/antibiotics9110806 33202755 PMC7696612

[20] Mendes G, Ramalho JF, Bruschy-Fonseca A, Lito L, Duarte A, Melo-Cristino J, Caneiras C. 2022. Whole-genome sequencing enables molecular characterization of non-clonal group 258 high-risk clones (ST13, ST17, ST147 and ST307) among carbapenem-resistant Klebsiella pneumoniae from a Tertiary University Hospital centre in Portugal. Microorganisms 10:416. doi:10.3390/microorganisms10020416 35208876 PMC8875758

[21] Mezzatesta ML, Caio C, Gona F, Cormaci R, Salerno I, Zingali T, Denaro C, Gennaro M, Quattrone C, Stefani S. 2014. Carbapenem and multidrug resistance in Gram-negative bacteria in a single centre in Italy: considerations on in vitro assay of active drugs. Int J Antimicrob Agents 44:112–116. doi:10.1016/j.ijantimicag.2014.04.014 25059444

[22] Özad Düzgün A. 2021. From Turkey: first report of KPC-3- and CTX-M-27-producing multidrug-resistant Klebsiella pneumoniae ST147 clone carrying OmpK36 and Ompk37 porin mutations. Microb Drug Resist 27:1265–1270. doi:10.1089/mdr.2020.0274 33794115

[23] López-Cerero L, Egea P, Gracia-Ahufinger I, González-Padilla M, Rodríguez-López F, Rodríguez-Baño J, Pascual A. 2014. Characterisation of the first ongoing outbreak due to KPC-3-producing Klebsiella pneumoniae (ST512) in Spain. Int J Antimicrob Agents 44:538–540. doi:10.1016/j.ijantimicag.2014.08.006 25446907

[24] EUCAST. 2023. Clinical breakpoints and epidemiological cut-off value. Available from: http://www.eucast.org/clinical_breakpoints

[25] Bortolaia V, Kaas RS, Ruppe E, Roberts MC, Schwarz S, Cattoir V, Philippon A, Allesoe RL, Rebelo AR, Florensa AF, et al.. 2020. ResFinder 4.0 for predictions of phenotypes from genotypes. J Antimicrob Chemother 75:3491–3500. doi:10.1093/jac/dkaa345 32780112 PMC7662176

[26] Neal-McKinney JM, Liu KC, Lock CM, Wu W-H, Hu J. 2021. Comparison of MiSeq, MinION, and hybrid genome sequencing for analysis of Campylobacter jejuni. Sci Rep 11:5676. doi:10.1038/s41598-021-84956-6 33707610 PMC7952698

[27] Alikhan N-F, Petty NK, Ben Zakour NL, Beatson SA. 2011. BLAST Ring Image Generator (BRIG): simple prokaryote genome comparisons. BMC Genomics 12:402. doi:10.1186/1471-2164-12-402 21824423 PMC3163573

[28] Sullivan MJ, Petty NK, Beatson SA. 2011. Easyfig: a genome comparison visualizer. Bioinformatics 27:1009–1010. doi:10.1093/bioinformatics/btr039 21278367 PMC3065679

[29] Li H, Durbin R. 2010. Fast and accurate long-read alignment with Burrows-Wheeler transform. Bioinformatics 26:589–595. doi:10.1093/bioinformatics/btp698 20080505 PMC2828108

[30] Li H, Durbin R. 2009. Fast and accurate short read alignment with Burrows-Wheeler transform. Bioinformatics 25:1754–1760. doi:10.1093/bioinformatics/btp324 19451168 PMC2705234

[31] Li H, Handsaker B, Wysoker A, Fennell T, Ruan J, Homer N, Marth G, Abecasis G, Durbin R. 1000. Genome Project Data Processing Subgroup. 2009. The sequence alignment/map format and SAMtools. Bioinformatics 25:2078–2079. doi:10.1093/bioinformatics/btp352 PMC2723002 19505943

[32] Carver T, Berriman M, Tivey A, Patel C, Böhme U, Barrell BG, Parkhill J, Rajandream M-A. 2008. Artemis and ACT: viewing, annotating and comparing sequences stored in a relational database. Bioinformatics 24:2672–2676. doi:10.1093/bioinformatics/btn529 18845581 PMC2606163

[33] Bragagnolo N, Audette GF. 2022. Solution characterization of the dynamic conjugative entry exclusion protein TraG. Struct Dyn 9:064702. doi:10.1063/4.0000171 36590369 PMC9797247

[34] Virolle C, Goldlust K, Djermoun S, Bigot S, Lesterlin C. 2020. Plasmid transfer by conjugation in Gram-negative bacteria: from the cellular to the community level. Genes (Basel) 11:1239. doi:10.3390/genes11111239 33105635 PMC7690428

[35] Chen LPG BPMMKBPJD. 2017. Genomic diversity and global epidemiology of carbapenem-resistant K. pneumoniae (CRKp) clonal group 147 (CG147), abstr 25. ASM Microbe, New Orleans, LA, USA

[36] Ding L, Shen S, Chen J, Tian Z, Shi Q, Han R, Guo Y, Hu F. 2023. Klebsiella pneumoniae carbapenemase variants: the new threat to global public health. Clin Microbiol Rev 36:e0000823. doi:10.1128/cmr.00008-23 37937997 PMC10732083

[37] Naas T, Cuzon G, Truong H-V, Nordmann P. 2012. Role of ISKpn7 and deletions in blaKPC gene expression. Antimicrob Agents Chemother 56:4753–4759. doi:10.1128/AAC.00334-12 22733068 PMC3421896

[38] Curiao T, Morosini MI, Ruiz-Garbajosa P, Robustillo A, Baquero F, Coque TM, Cantón R. 2010. Emergence of blaKPC-3-Tn4401a associated with a pKPN3/4-like plasmid within ST384 and ST388 Klebsiella pneumoniae clones in Spain. J Antimicrob Chemother 65:1608–1614. doi:10.1093/jac/dkq174 20513703

[39] Elias R, Spadar A, Hendrickx APA, Bonnin RA, Dortet L, Pinto M, Phelan JE, Portugal I, Campino S, da Silva GJ, Clark TG, Duarte A, Perdigão J. 2023. Emergence of KPC-3- and OXA-181-producing ST13 and ST17 Klebsiella pneumoniae in Portugal: genomic insights on national and international dissemination. J Antimicrob Chemother 78:1300–1308. doi:10.1093/jac/dkad093 36999363

[40] Nordmann P, Naas T, Poirel L. 2011. Global spread of carbapenemase-producing Enterobacteriaceae. Emerg Infect Dis 17:1791–1798. doi:10.3201/eid1710.110655 22000347 PMC3310682

[41] Nordmann P, Cuzon G, Naas T. 2009. The real threat of Klebsiella pneumoniae carbapenemase-producing bacteria. Lancet Infect Dis 9:228–236. doi:10.1016/S1473-3099(09)70054-4 19324295

