# Erratum for Recacha et al., “Characterization of a *bla*_KPC-3_-carrying plasmid in a clinical isolate of *Klebsiella pneumoniae* belonging to the emerging successful clone ST147”

**DOI:** 10.1128/spectrum.00408-26

**Published:** 2026-03-20

**Authors:** Esther Recacha, Mercedes Delgado-Valverde, Marina R. Pulido, Elena Pérez-Nadales, Patricia Pérez-Palacios, Inés Portillo-Calderón, Juan Manuel Sánchez-Calvo, Irene Gracia-Ahufinger, Álvaro Pascual

**Affiliations:** 1 Unidad de Gestión Clínica de Enfermedades Infecciosas y Microbiología, Hospital Universitario Virgen Macarena 16582, Seville, Spain; 2 Instituto de Biomedicina de Sevilla/Hospital Virgen Macarena, Universidad de Sevilla/CSIC 16778, Seville, Spain; 3 Centro de Investigación Biomédica en Red en Enfermedades Infecciosas (CIBERINFEC), Instituto de Salud Carlos III 38176, Madrid, Spain; 4 Departamento de Microbiología, Facultad de Medicina, Universidad de Sevilla 16778, Seville, Spain; 5 Instituto Maimónides de Investigación Biomédica de Córdoba (IMIBIC), Hospital Universitario Reina Sofía 16501, Córdoba, Spain; 6 Departamento de Química Agrícola, Edafología y Microbiología (área Microbiología), Universidad de Córdoba 27987, Córdoba, Spain; 7 Unit of Infectious Diseases and Clinical Microbiology, Jerez de la Frontera University Hospital 16844, Jerez de la Frontera, Spain; 8 Microbiology Unit, Hospital Universitario Reina Sofía 16501, Córdoba, Spain

## ERRATUM

Volume 13, no. 7, e02338-24, 2025, https://doi.org/10.1128/spectrum.02338-24. The affliations and affiliation letters for each author should appear as shown in this erratum.

