# Supplementary material for: Characterization of a blaKPC-3-carrying plasmid in a clinical isolate of Klebsiella pneumoniae belonging to the emerging successful clone ST147
Source: Microbiol Spectr. 2025 May 23;13(7):e02338-24. doi: 10.1128/spectrum.02338-24 (PMC12211030; doi:10.1128/spectrum.02338-24)
Supplement: Fig. S1 — Dendrogram of pulsed-field gel electrophoresis analysis. [file spectrum.02338-24-s0001.docx]

**Supplemental material**

**Figure S1**. Dendrogram of pulsed-field gel electrophoresis (PFGE) analysis of *Xba*I-digested DNA illustrating genetic relationships between the *Klebsiella pneumoniae* ST147/KPC-3 isolates. The dendrogram was created with Bionumerics 8.1 software (Applied Maths, Marcy-l’Etoile, France) using the Dice coefficient with position tolerance settings of 1% optimization and 0.8% band position tolerance.

**
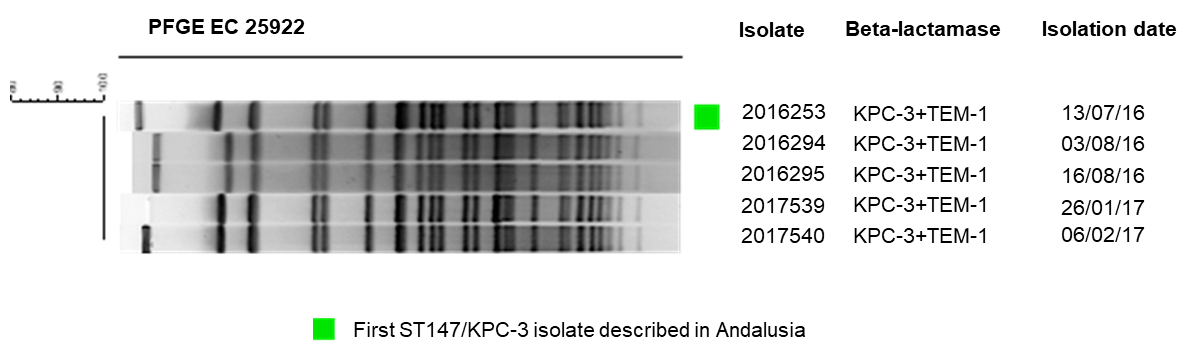
**
